# Presence and Implications of Sarcopenia in Non-alcoholic Steatohepatitis

**DOI:** 10.3390/metabo11040242

**Published:** 2021-04-15

**Authors:** Gregory Habig, Christa Smaltz, Dina Halegoua-DeMarzio

**Affiliations:** 1 Department of Medicine, Thomas Jefferson University, Philadelphia, PA 19107, USA; Gregory.Habig@jefferson.edu (G.H.); christa.smaltz@jefferson.edu (C.S.); 2 Department of Medicine, Division of Gastroenterology and Hepatology, Thomas Jefferson University, Philadelphia, PA 19107, USA

**Keywords:** non-alcoholic fatty liver disease (NAFLD), non-alcoholic steatohepatitis (NASH), sarcopenia, metabolic syndrome (MS), cirrhosis

## Abstract

Sarcopenia, defined as the loss of muscle strength, mass, and functionality, confers a poor prognosis in the setting of cirrhosis. Given its clinical significance, a better understanding of the underlying mechanisms leading to cirrhosis, sarcopenia, and their co-occurrence may improve these patients’ outcomes. Non-alcoholic steatohepatitis (NASH) shares many of the same etiologies as sarcopenia, including insulin resistance, chronic inflammation, and ectopic adipocyte deposition, which are hallmarks of metabolic syndrome (MS). NASH thus serves as a prime candidate for further exploration into the underlying pathophysiology and relationship between these three conditions. In this review, we discuss the natural history of NASH and sarcopenia, explore the interplay between these conditions in the scope of MS, and seek to better define how an assessment of muscle mass, strength, and functionality in this population is key to improved diagnosis and management of patients with sarcopenia and NASH.

## 1. Introduction

Mirroring the increase in the incidence of obesity and metabolic syndrome, cases of non-alcoholic fatty liver disease (NAFLD) have seen a significant uptick over the past several decades. In fact, the global prevalence of NAFLD is now estimated to include 25% of the total population with liver disease [1], and it afflicts up to 70–90% of patients in high-risk groups like those with obesity, type II diabetes mellitus (TIIDM), and metabolic syndrome (MS) [2]. Unsurprisingly, the United States has been far from immune to this trend, with NAFLD occupying an increasing proportion of the causes of cirrhosis in America. According to 2011 estimates, NAFLD accounted for about 75% of chronic liver disease in the United States [3]. Given this trend toward a growing at-risk population, a better understanding of risk factors for rapid deterioration in end-stage liver disease (ESLD) is necessary as the number of patients requiring transplant will inevitably increase without a compensatory rise in available transplant organs.

In the current landscape of liver transplantation, the Model for End-stage Liver Disease (MELD) is used to prioritize patients on the transplant list. This measure continuously evolves to include new factors which garner a patient more points to move them toward the top of the transplant list, such as those gained with hepatocellular carcinoma (HCC) within the Milan criteria, or the presence of porto-pulmonary hypertension of a given severity. Despite the impact of such risk factors for clinical deterioration while awaiting transplant, this classification still excludes key factors associated with increased mortality and post-operative outcomes such as sarcopenia [4].

Sarcopenia is defined as a reduction in muscle mass, strength, and overall functionality. In the setting of advanced liver disease, this term is used to describe the loss in muscle mass that occurs with cirrhosis and has been identified as a serious risk factor for clinical deterioration and poor prognosis [5]. This loss of functional capacity has been the target of research recently due to its association with increased morbidity, mortality, and healthcare expenditure [6]. This review seeks to compile the existing literature on the subject and more completely identify the most appropriate definition and mode of diagnosing sarcopenia to better classify the risk factor in patients with cirrhosis. Furthermore, through a review of their shared association with MS and underlying pathophysiology leading to each condition, this review may elucidate additional lines of inquiry to better investigate and understand the interplay between sarcopenia and NAFLD.

## 2. Metabolic Syndrome and NASH

MS is described as an asymptomatic combination of physiologic abnormalities characterized by insulin resistance, increased abdominal girth, low HDL cholesterol, abnormally elevated triglycerides, blood pressure, and fasting blood glucose [7]. Clinically this syndrome is defined by diagnoses of conditions such as hyperlipidemia (HLD), essential hypertension (HTN), and TIIDM. Despite its absence from the diagnostic criteria, NAFLD has been found to have a significant association with conditions in metabolic syndrome. A recent meta-analysis on the subject demonstrated that over 80% of patients with NASH, a condition under the umbrella of NAFLD, had an elevated BMI. Furthermore, in this group, 72% of patients had HLD, and 44% had impaired fasting plasma glucose consistent with TIIDM [1]. Despite the fact that the association between these two syndromes has been well established, the pathophysiological mechanisms relating the two are still under investigation. 

When first investigated, the pathogenesis of NASH was described by a two-hit hypothesis: the first hit causing the appearance of steatosis, and the second causing inflammation, damage to hepatocytes, and eventually fibrosis [8]. More recently, however, another hypothesis, the multi-parallel hypothesis, has been used to describe the pathogenesis of NASH. This hypothesis theorizes that NASH arises from multiple processes acting in concert with one another, such as oxidative stress, abnormal lipid metabolism, lipotoxicity, and abnormal cytokine production leading to eventual steatosis, hepatitis, and fibrosis [9]. Similarly, the pathogenesis of metabolic syndrome has been theorized to be multifactorial, with lipid and glucose homeostasis issues affecting insulin-sensitive tissues such as the liver, muscles, and adipocytes [10]. As a result of their shared pathophysiology, specifically related to lipid metabolism, it comes as no surprise that metabolic syndrome has such clear and deleterious effects on the liver. Likewise, it stands to reason that metabolic syndrome would have equally harmful effects on other insulin-sensitive tissues such as skeletal muscle.

## 3. Metabolic Syndrome and Sarcopenia

Although there are many different underlying risk factors and etiologies that can lead to the development of sarcopenia, its relationship to metabolic syndrome has become of increasing interest. Recent evidence suggests an increased risk for cardiovascular events, TIIDM, and other adverse outcomes when sarcopenia is seen with one or more features of metabolic syndrome [11,12]. One of the main cornerstones of metabolic syndrome is increased insulin resistance. Many studies have examined the relationship between sarcopenia and insulin resistance and noted a complex interplay between the two conditions. It has been estimated that the prevalence of sarcopenia in patients with TIIDM is as high as 15%, and the two variables have been independently associated when linear regressions were performed [13]. The mechanism for their connection remains unclear, but it has been postulated that the low levels of inflammation present with TIIDM could lead to dysregulated muscle homeostasis. Patients with increased insulin resistance have been found to not only have a greater risk of losing their lean body fat, but poor glycemic control has also been correlated with decreased physical performance [14,15]. Conversely, low lean muscle mass has been correlated to an increased risk of TIIDM and insulin resistance. Skeletal muscle, as one of the primary deposits for insulin-mediated glucose disposal, accounts for approximately 80% of glucose clearance [16,17]. Thus, with the loss of skeletal muscle, studies have conferred an increased risk of developing TIIDM [18,19].

Obesity, another cornerstone of metabolic syndrome, has also been linked to sarcopenia. The term sarcopenic obesity refers to the loss in skeletal muscle mass accompanied by the gain in visceral adipose tissue. This combination is especially important because the two conditions in concert have been shown to increase the risk of physical disability when compared to either sarcopenia or obesity alone [20]. This relationship seems to be bidirectional as well, with the loss of lean muscle mass leading to decreased physical activity and thus increasing the risk of obesity. Accumulation of visceral adipose tissue can also lead to sarcopenia by directly downregulating contractile proteins necessary for skeletal muscle cells’ proper functioning [21]. 

Chronic inflammation, another factor in the pathogenesis of metabolic syndrome, can be closely associated with sarcopenia. Interleukin 6 (IL-6), an inflammatory cytokine that circulates in the bloodstream in many disease states, has been studied for its effects on skeletal muscle. One animal study infused a low level of IL-6, comparable to levels seen chronically in older individuals, into a targeted muscle in mice to examine its direct effects on skeletal muscle. This study showed that muscles exposed to IL-6 atrophied and resulted in a catabolic signaling pattern in these exposed cells [22]. Another longitudinal study looked at the effects of IL-6 and C-reactive protein (CRP) on total appendicular skeletal muscle (TASM) loss in non-sarcopenic adults. They found that increased levels of IL-6 and CRP correlated to an increased risk of loss of TASM over a 5year period [23]. An increased level of these inflammatory cytokines, including TNF-alpha, has also been shown to negatively impact both muscle mass and function [24,25].

**Figure 1:**
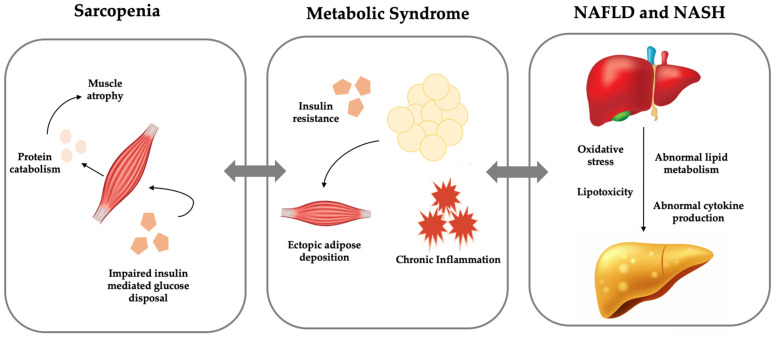
Visual representation of interplay between metabolic syndrome, non-alcoholic fatty liver disease (NAFLD), and sarcopenia.

Although obesity, chronic inflammation, and increased insulin resistance have each individually been linked to sarcopenia, it appears that all three are intimately connected and occur synergistically to cause deterioration in functional status and increased morbidity and mortality. While the pathway by which metabolic syndrome and sarcopenia are related is not entirely known, it is thought to be due to the complex interplay of inflammation, fat deposition, and insulin resistance. This combination ultimately leads to the loss of skeletal muscle mass and function. Because obese individuals have a higher proportion of visceral adipose tissue and therefore more adipocytes, which secrete high levels of inflammatory cytokines, these patients’ muscle tissue is in a perpetual state of inflammation, increasing the risk for muscle atrophy [23,26]. Furthermore, obesity leads to ectopic lipid storage in skeletal muscle, causing both impaired skeletal muscle activation and increased risk for insulin resistance in the tissue [21,27,28] (Figure 1). Combining these processes in a patient with obesity results in a significant challenge that their skeletal muscle cannot tolerate indefinitely, culminating in the poor functional outcomes detailed above.

## 4. Non-alcoholic Steatohepatitis (NASH) and Sarcopenia

Given their shared pathophysiology, it comes as no surprise that the interplay between NASH and sarcopenia has been frequently investigated [29,30,31,32,33,34,35,36]. The chronic inflammation and ectopic fat deposition discussed above has been found to have clear and deleterious effects on patients with the condition. While these underlying connections to MS serve as a likely primary driver of pathologic functioning of hepatocytes and myocytes, they are not the only theorized stimuli in the development of sarcopenia in NASH. More specifically, the lack of readily available glycogen stores in cirrhotic livers is thought to make glycogenolysis a challenge for patients with NASH. As a result, there is upregulation of gluconeogenesis primarily via the breakdown of amino acids in muscle tissue. In combination with poor oral intake commonly observed in patients with cirrhosis, this results in net catabolism with more significant protein breakdown than production and thus the development of sarcopenia [37]. The decrease in muscle mass observed in sarcopenia, in turn, leads to a smaller proportion of skeletal muscle participating in insulin-mediated glucose disposal, resulting in insulin resistance. The increase in insulin resistance subsequently results in an upregulation of lipolysis and increased fatty acid delivery to and deposition in muscle and liver tissue, causing worsened myosteatosis and hepatosteatosis [32]. Some evidence even suggests that myosteatosis may precede sarcopenia in patients with NASH. More precisely, one animal study showed that severity of fatty infiltration into muscle tissue reflected hepatosteatosis severity in early NASH, even when sarcopenia was not observed [38]. Conversely, normal secretion of several skeletal muscle cytokines and myokines, such as IL-6 and irisin, has been shown to be protective from hepatosteatosis by promoting β-oxidation [32]. However, pathological secretion of these factors, increased release of IL-6, and decreased availability of irisin diminishes the protective effects, thus increasing the risk of developing NASH [32]. Combined, the negative effects of MS, patients’ hyper-catabolic state, and poor enteral intake in cirrhosis leaves skeletal muscle little opportunity to sustain its structure and function in NASH, promoting further fat deposition into hepatic tissue. 

**Figure 2:**
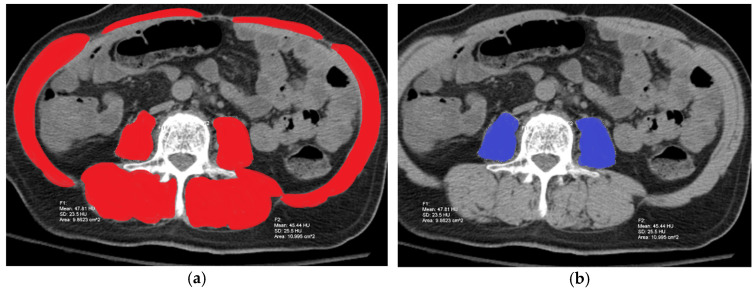
CT scan of a 68-year-old male patient at L3 demonstrating how sarcopenia is assessed based on measurement of SMI in red (**a**) as defined by cross-sectional areas of psoas, erector spinae, quadratus lumborum, transversus abdominis, rectus abdominis, and internal and external obliques, and measurement for psoas muscle area (PMA) in blue (**b**) as measured by the sum of the areas of the psoas alone.

Irrespective of the exact cause, a clear consensus on how sarcopenia should be defined in cirrhosis and how it is investigated is still a topic of discussion. The most widely accepted measure of sarcopenia in cirrhosis in the United States, as defined by the North American consensus statement, is the Skeletal Muscle Index (SMI) as measured on CT [39]. This recommendation is largely based on a 2017 multicenter retrospective study looking at wait list mortality in patients based on pretransplant sarcopenia. This study established the cutoff values of SMI at the L3 vertebrae of <50 cm^2^/m^2^ in men and <39 cm^2^/m^2^ in women based on their results showing significantly higher mortality leading up to transplant in patients with SMI below these values (Figure 2a) [31]. Based on this strong evidence, the European Association for the Study of the Liver (EASL) and the Japan Society of Hepatology have also recommended SMI as the measure of choice for diagnosing sarcopenia; however, the exact cutoff values for diagnosing the condition differ slightly [40,41]. This measure is not accepted by all as the gold standard for appropriately assessing sarcopenia in ESLD, in part because it does not account for measures of skeletal muscle function. More specifically, functional measurements such as handgrip strength testing (HGT), chair stand test (CST), and the six-minute walk test have also shown to be significant prognostic indicators of mortality in ESLD [5,32]. Despite this association, functional measures of sarcopenia are largely overlooked in clinical practice. As it currently stands, the differences in opinion for measuring sarcopenia in cirrhosis are so vast that in a 2016 meta-analysis and review on the subject, seven different approaches to measuring sarcopenia in cirrhosis were used in the 19 studies reviewed [42]. Within the scope of cross-sectional imaging, other measures such as psoas muscle area (PMA) (Figure 2b) and transverse psoas muscle thickness (TPMT) have also shown some promise as simple measures to diagnose sarcopenia but have yet to offer significant enough evidence to replace SMI [43,44]. Other less invasive modalities have also been investigated to assess sarcopenia in cirrhosis, such as DEXA, ultrasound, and biomedical impedance, with varying results, but none have proven as clinically significant a tool in screening for sarcopenia as CT [5,45,46].

### 4.1. Clinical Significance of Sarcopenia in NASH

An improved definition of the measurements of sarcopenia is of clinical interest as sarcopenia has been associated with acute decompensation of chronic liver disease, increased one- and five-year mortality post-transplant, as well as overall mortality in patients with cirrhosis [47,48]. This is especially pertinent to NASH, as one study found a direct correlation between sarcopenia as defined by weight-adjusted SMI, and histologic progression of NASH to cirrhosis [29]. This finding was corroborated by a subsequent meta-analysis that suggested a direct relationship between sarcopenia and NAFLD [30]. Given its significant effect on mortality, poor post-transplant outcomes, and progression of NASH to cirrhosis, it could be suggested that sarcopenia may hold utility at some point as a marker for clinical deterioration in earlier stages of NAFLD. This could be an extremely useful clinical marker, especially if sarcopenia can be detected at a point early enough to intervene and prevent further progression. Given that sarcopenia has been clearly linked with increased mortality in liver patients, one study even looked at incorporating a measurement of sarcopenia along with the MELD criterion to help prognosticate mortality in patients with cirrhosis [5]. As the prevalence of NAFLD and NASH-related mortality continues to rise in the United States, targeting more at-risk patients for progression to cirrhosis along with cirrhosis-related mortality could help combat the exponential rise in NASH-related mortality. Predictive modeling suggests a 168% increase in patients with decompensated cirrhosis and a 178% increase in liver-related deaths by 2030 [49]. A reliable method to detect sarcopenia in these patients could improve outcomes in liver transplant patients and help build more targeted, comprehensive therapy for patients with a condition on the spectrum of NAFLD. 

### 4.2. Management of Sarcopenia in NASH

Although surgical and medical interventions exist in the management of sarcopenia in NASH, the primary treatment remains lifestyle modification. While a change in diet is recommended, it is important to consider nutritional status, as patients with both NASH and sarcopenia can have wide variability in body habitus. To be more precise, some patients with NASH may have sarcopenic obesity, with a high BMI but overall low muscle mass. Conversely, they may also have a normal to low BMI in the setting of inadequate oral intake and increased muscle catabolism. As a result, while the former condition may improve from caloric restriction, the latter could lead to further loss in muscle mass [50]. To address these differing nutritional requirements, dietary recommendation focuses on a high-protein, low-fat, and low-starch diet with frequent snacking to combat the catabolic state associated with fasting in cirrhosis [51,52]. Moderate exercise has also been shown to stimulate muscle growth, and while more investigation is needed to determine the exact type and regimen of activity that is best suited to improve sarcopenia, exercise remains an important aspect of the management of the condition [50]. One interesting test to assess the response to these interventions is the six-minute walk test, a functional measure of cirrhosis that was alluded to earlier. This test has not only been shown to be suggestive of wait list mortality, with patients who fail to walk greater than 520m meeting the definition for sarcopenia and having increased mortality, but also may be a useful clinical tool, as the mortality estimate in these patients is cut in half with every 100m improvement in test performance [53]. 

Although lifestyle modifications are the main focus of therapy for sarcopenia in NASH, medical and surgical interventions are attempted as well. Medical therapies primarily focus on addressing the predisposing conditions to developing NASH and MS, such as HDL and TIIDM. There are no current FDA-approved pharmacologic therapies directly for NASH and sarcopenia in the United States. Surgical interventions are similarly limited. While there is some evidence suggesting that sarcopenia can improve in as much as 28% of patients following a liver transplant, these results are likely confounded by post-transplant complications and other comorbid conditions [47]. Additional investigation into the effects of a liver transplant on sarcopenia in patients with NASH cirrhosis is necessary to better understand how a transplant may benefit these patients from a frailty perspective and determine if there are any changes in long-term outcomes. 

## 5. Conclusion

NASH and MS, given their similar pathophysiology, seem inextricably linked. The loss in muscle mass and function that occurs in concert with these two conditions helps perpetuate the increased incidence of morbidity and mortality seen in these patients. Through an exploration of these conditions and their co-occurrence, it becomes clear that insulin resistance, chronic inflammation, and a tendency toward catabolism play central roles in fatty infiltration of the liver, development of MS, and the loss of skeletal muscle structure and function characteristic of sarcopenia. This investigation highlights that while the generally accepted measure for assessing sarcopenia in cirrhosis has been well defined, understanding the clinical significance of the condition in cirrhosis requires further investigation and may necessitate a multi-modal approach. More specifically, the combination of cross-sectional imaging and functional measures of sarcopenia in cirrhosis may provide the best measure of clinically significant muscle loss in cirrhosis. This combination of structural and functional measures of sarcopenia, in addition to a better understanding of the underlying pathophysiology contributing to the conditions’ development in cirrhosis, may also enable a more targeted approach to its treatment. Through this discussion, it has become clear that additional investigation into the best imaging modality, measure, and treatment is needed to aid in the diagnosis and treatment of sarcopenia in cirrhosis. Sarcopenia in NASH requires continued evaluation and inclusion into prognostic scores like the MELD, given the increasing body of evidence suggesting its clinical significance in predicting poor outcomes in the patients it afflicts.
